# Batch-effect correction in single-cell RNA sequencing data using JIVE

**DOI:** 10.1093/bioadv/vbae134

**Published:** 2024-09-13

**Authors:** Joseph Hastings, Donghyung Lee, Michael J O’Connell

**Affiliations:** Department of Statistics, Miami University, Oxford, OH 45056, United States; Department of Statistics, Miami University, Oxford, OH 45056, United States; Department of Statistics, Miami University, Oxford, OH 45056, United States

## Abstract

**Motivation:**

In single-cell RNA sequencing analysis, addressing batch effects—technical artifacts stemming from factors such as varying sequencing technologies, equipment, and capture times—is crucial. These factors can cause unwanted variation and obfuscate the underlying biological signal of interest. The joint and individual variation explained (JIVE) method can be used to extract shared biological patterns from multi-source sequencing data while adjusting for individual non-biological variations (i.e. batch effect). However, its current implementation is originally designed for bulk sequencing data, making it computationally infeasible for large-scale single-cell sequencing datasets.

**Results:**

In this study, we enhance JIVE for large-scale single-cell data by boosting its computational efficiency. Additionally, we introduce a novel application of JIVE for batch-effect correction on multiple single-cell sequencing datasets. Our enhanced method aims to decompose single-cell sequencing datasets into a joint structure capturing the true biological variability and individual structures, which capture technical variability within each batch. This joint structure is then suitable for use in downstream analyses. We benchmarked the results against four popular tools, Seurat v5, Harmony, LIGER, and Combat-seq, which were developed for this purpose. JIVE performed best in terms of preserving cell-type effects and in scenarios in which the batch sizes are balanced.

**Availability and implementation:**

The JIVE implementation used for this analysis can be found at https://github.com/oconnell-statistics-lab/scJIVE.

## 1 Introduction

There have been significant advancements in recent years in single-cell RNA sequencing (scRNA-seq) (Luecken and Theis 2019). Single-cell sequencing provides a higher resolution view of genomic data when compared to bulk RNA sequencing (bulk RNA-seq) and allows for the heterogeneity of different cell populations to be preserved. Bulk RNA-seq measures the average level gene expression in a population of cells (Wang *et al*. 2009), while scRNA-seq measures the gene expression for each cell individually (Wang and Navin 2015). Another prominent feature in scRNA-seq data is the high proportion of zero counts (Vallejos *et al*. 2017). This is due to both biological and technical reasons. One biological cause of this zero-inflation could be due to a particular cell type having very little or no gene expression. A technical cause could be due to what is referred to as a technical dropout, where a certain gene is expressed but does not get detected by the sequencing technology. Multiple sequencing protocols have been developed to capture this information, with prominent examples including CEL-seq2, Drop-seq, MARS-seq, SCRB-seq, Smart-seq, and Smart-seq2 (Ziegenhain *et al*. 2017). Sequencing data consists of a count matrix for a given set of genes obtained from a sample of cells. These counts represent the number of times that a specific gene is detected in a single cell (Grün and van Oudenaarden 2015). Each row represents a gene and each column is a cell. The library size for a cell is the sum of all counts across all genes.

Once the count data are obtained, it is typically normalized to help account for any variability caused by sampling effects within the given sequencing protocol (Grün and van Oudenaarden 2015). A few examples of normalization methods include counts per million (CPM) normalization, upper quartile (UQ) normalization, and trimmed mean of M values (TMM) normalization (Vallejos *et al*. 2017). In CPM normalization, each count is divided by its cell’s library size and multiplied by a million. UQ normalization uses a scaling factor proportional to the 75th percentile of the counts for a given cell. TMM normalization seeks to trim away cell counts that exhibit large log-fold differences within a cell.

Another common preprocessing step is the integration of multiple datasets that are obtained from different batches. Unwanted technical variation and differences between count data are known as batch effects (Zhang *et al*. 2020). These effects can arise due to different sequencing technologies being used or cells being sequenced at different times.

### 1.1 Batch-effect correction/data integration methods

Traditional batch effect correction methods, including Supervised Surrogate Variable Analysis (SVA; Leek 2014), Unsupervised SVA (Leek and Storey 2008), Independent SVA (Teschendorff *et al*. 2011), Removing Unwanted Variation (Gagnon-Bartsch and Speed 2012, Risso *et al*. 2014), and ComBat (Johnson *et al*. 2007)/ComBat-seq (Zhang *et al*. 2020), are primarily developed for bulk RNA-seq datasets. These methods are designed to identify and mitigate hidden unwanted variations, such as batch effects, effectively distinguishing biological signals from noise. Utilizing dimension reduction techniques such as Principal Component Analysis (PCA), Singular Value Decomposition (SVD), and Independent Component Analysis, they aim to preserve the integrity of biological signals, thus enhancing the reliability of differential expression analyses between conditions. They achieve this by inferring orthogonal transformations of hidden non-biological factors that can be used as covariates in downstream analysis.

Transitioning to single-cell sequencing, a range of methods have been implemented to address the unique challenges of these data. Notable among these are single-cell latent variable model (scLVM) (Buettner *et al*. 2015), Mutual Nearest Neighbors (MNNs) (Haghverdi *et al*. 2018), Harmony (Korsunsky *et al*. 2019), Seurat v5 (Stuart *et al*. 2019), and LIGER (Liu *et al*. 2020, Welch *et al*. 2019), which are recognized for their advanced integration capabilities, scalability, and sophisticated visualization tools. Seurat v5 and LIGER, in particular, adept at aligning multiple datasets across different conditions, technologies, or species, using methods like reciprocal PCA and integrative non-negative matrix factorization to preserve biological variation while mitigating non-biological hidden heterogeneity. This stands in contrast to traditional batch correction methods such as ComBat-seq, which primarily focus on simply correcting for batch effects.

A reasonable alternative to the above methods would be to use a multi-source dimension reduction method, such as joint and individual variation explained (JIVE) (Lock *et al*. 2013). JIVE decomposes two or more biological datasets into three low-rank approximation components: a joint structure among the datasets, individual structures unique to each distinct dataset, and residual noise. The goal in this context would be to use the individual structure to identify the batch effects and the joint structure to identify the biological effects. In this study, we will benchmark Seurat v5, Harmony, LIGER, and ComBat-seq, comparing the performance of JIVE (with computational improvements to be more scalable for single-cell sequencing data) against these integration methods.

#### 1.1.1 Seurat v5

Seurat v5 is software package developed by the Satija Lab, which provides a comprehensive set of tools for single-cell data analysis and integration (Stuart *et al*., 2019). The Seurat v5 integration method builds upon their previous work by leveraging a new graph-based approach. First, log-normalization is performed on all datasets and expression values are standardized for each gene. A subset of features is selected, which exhibits high variance across all datasets. Then an initial dimension reduction method utilizing canonical correlation analysis is performed to ensure similarities across datasets are preserved. Canonical correlation vectors are then approximated and used to identify K-nearest neighbors (KNNs) for each cell within their paired dataset. MNNs are then identified to act as anchors between datasets. These anchors are then filtered, scored, and weighted using the new shared nearest neighbors (SNN) approach, and finally used to perform the batch correction.

#### 1.1.2 Harmony

Harmony is an integration method designed to model and eliminate effects of known sources of variation (Korsunsky *et al*. 2019). The method utilizes with an initial low-dimensional representation of the data, such as principal components, and then iterates between two algorithms until convergence is reached. The first algorithm clusters cells from multiple batches, but ensures that the diversity of batches within each cluster is maximized (i.e. maximum diversity clustering). The second algorithm then uses a mixture model-based approach to perform linear batch correction from a given vector of the known batches. The clustering step assigns soft clusters to cells and the correction step uses these clusters to compute new cell embeddings from the previous iteration.

### 1.2 Joint and individual variation explained

JIVE was originally created for integrating different types of bulk sequencing data. For example, Lock *et al*. (2013) used gene expression and miRNA data from a set of 234 Glioblastoma Multiforme tumor cells in an attempt to identify any joint or individual variation between the data types. JIVE can be expressed in terms of PCA, a common dimension reduction technique. In PCA, given a dataset *X* of *p* genes by *n* cells, we can calculate a rank *r* approximation of *X* in the following way:

(1) $$X_{p\times n}\;\approx U_{p\times r}S_{r\times n}$$

In Equation (1), $U_{p\times r}$ is the first *r* columns from the loading matrix and $S_{r\times n}$ is the first *r* rows from the scores matrix. The *i*-th row of $S_{r\times n}$ is called the *i*-th principal component of *X*, and each principal component is constructed to be orthogonal to all others. Since each principal component is orthogonal, the scores do not suffer from any issues due to multicollinearity.

The JIVE algorithm was initially designed for vertical integration, where different types of omics data that measure the same biological entity are combined (e.g. DNA, RNA, protein, etc.; Chen and Snyder 2013). In this paper, we use horizonal integration, where different biological entities share the same gene set, so this notation differs slightly from Lock *et al*. (2013). Additionally, to do horizontal integration, we need to transpose the data, so each $X_{i}^{T}$ will be $n_{i}$ cells by *p* genes. For *k* batches, the JIVE decomposition can be written in the following manner:

(2) $$\begin{array}{c} X_{1}^{T}\approx U_{1}S+W_{1}S_{1}+R_{1}\\ X_{2}^{T}\approx U_{2}S+W_{2}S_{2}+R_{2}\\ \vdots \\ X_{k}^{T}\approx U_{k}S+W_{k}S_{k}+R_{k} \end{array}$$

We will denote $n_{1}+n_{2}+\cdots +n_{k}=n$. Let *r* denote the chosen rank for the joint structure and $r_{i}$ denote the chosen individual ranks for each $X_{i}^{T}$. The matrix *U* of size n×r is equal to $\left({\begin{array}{cccc} U_{1}^{T} & U_{2}^{T} & \cdots & U_{k}^{T} \end{array}}^{T}\right)$, where $U_{i}$ are the scores of the joint structure for the individual datasets $X_{1}$, …, $X_{k}$, and *S* is an r×n loading matrix. The $W_{i}$ are $n_{i}\times r_{i}$ score matrices and the $S_{i}$ are $r_{i}\times p$ loading matrices for each $X_{i}$. The $R_{i}$ represent any remaining residual noise. The JIVE decomposition enforces orthogonality between the joint and individual structures, which ensures that the two structures capture distinct directions of variation within the data. This is important for ensuring that adjusting for batch effects does not result in the removal of important biological effects. For batch correction, the estimated joint components are used to generate the corrected count matrix ${\left({\hat{U}}_{k}\hat{S}\right)}^{T}$.

The JIVE decomposition estimates the joint and individual structures by minimizing the sum of squared error of the residual matrix. Given an initial estimate for the joint structure, it finds the individual structures to minimize the sum of squared error. Then, given the new individual structures, it finds a new estimate for the joint structure, which minimizes the sum of squared error. This process is repeated until a given threshold for convergence is reached. The existing implementation allows ranks to be estimated by one of two different methods: a permutation test rank selection and a Bayesian Information Criterion rank selection (O’Connell and Lock 2016). In this paper, we used the permutation test for rank selection, which is detailed in Lock *et al*. (2013).

While JIVE’s current implementation (O’Connell and Lock 2016) excels at integrating bulk sequencing data from different genomic layers, it is not feasible for integrating large-scale single-cell sequencing datasets, especially those from multiple batches. This incompatibility arises for two main reasons. First, single-cell data often has a substantially larger sample size than previous JIVE applications (i.e. bulk sequencing). Given this magnitude, necessary optimizations are required to mitigate the method’s computational burden. The current R-based implementation also contributes to this computational inefficiency. Second, JIVE was originally constructed for use as a vertical integration method. In this case, our data shares the same feature space (scRNA-seq), but each batch contains different biological entities. However, JIVE has previously been used for horizontal integration in meta-analysis Kim *et al*. (2018).

To address these challenges, we improved the computational efficiency of the JIVE algorithm by restructuring it using Rcpp and C++. Additionally, we utilized partial SVD to calculate the leading singular vectors with greater efficiency. Considering that all batches were sequenced over the same gene set, we can leverage JIVE as a horizontal integration method, using the shared variable set to match the batches. We incorporated these modifications within our updated software package. As a result, the new JIVE method can now estimate the joint structure matrix that captures the shared biological structure between scRNA-seq data from different batches and the individual structures that capture technical effects. We used the joint structure as the batch-corrected data, which allowed us to simultaneously perform dimension reduction and eliminate batch effects. To assess the performance of the enhanced JIVE for scRNA-seq data batch-effect correction, we benchmarked it against two established methods: Seurat v5 and Harmony, using both simulated and real scRNA-seq datasets. The efficacy of each method was evaluated using five distinct batch correction evaluation metrics.

## 2 Methods

### 2.1 JIVE algorithm improvements

The JIVE algorithm is currently implemented in the R.JIVE R package (O’Connell and Lock 2016). However, the functions provided in this package can take a substantial amount of runtime to get results (taking upwards of 12+ h depending on data). We improved the speed of these base functions in two main ways: utilizing partial SVD in the RSpectra R package (Qiu and Mei 2022) and converting frequently used matrix operations into precompiled C++ code using the Rcpp R package (Eddelbuettel and François 2011) and the RcppEigen R package (Bates and Eddelbuettel 2013), which provides access to the Eigen C++ linear algebra library.

The original R.JIVE code utilizes SVDs in many different areas; however, only the largest singular values/vectors are used. A full decomposition takes a lot of time and resources to compute, and the majority of the output is not used. We switched to using a partial SVD function in the RSpectra R package, which returns the largest singular values/vectors of a given matrix. We compared the partial SVD function to the base SVD function that is used in the R.JIVE package. A benchmark was performed on a dataset of size 1000×1000 generated from a standard normal distribution in which each function call was repeated 100 times. The top 1, 5, and 10 singular values/vectors were computed from each function call.

The other area in which we made significant improvements was in basic matrix operations. We tested two different functions which implemented C++ code to perform matrix multiplication and compared their performance to the default %*% operator in R. One function uses the Armadillo C++ library via the RcppArmadillo R package (Eddelbuettel and Sanderson 2014) and the other uses the Eigen C++ library via the RcppEigen R package. The function using RcppEigen allows us to specify the number of central processing unit (CPU) cores to utilize when performing computations. We compared the default matrix multiplication operator %*% in R to the implementations in RcppArmadillo and RcppEigen. A benchmark was performed by multiplying two matrices *A* and *B* of size 1000×1000 generated from a standard normal distribution in which each function call was performed 100 times.

**Figure 1.**
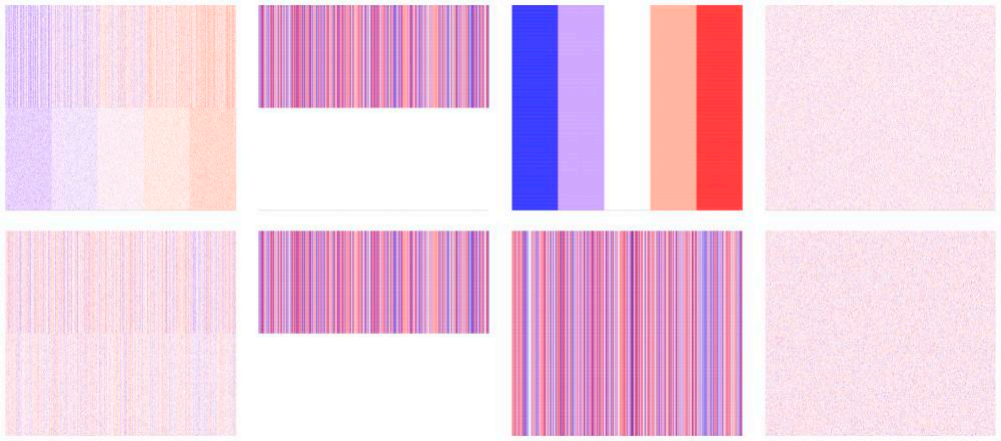
Heatmaps for simulation data used in JIVE benchmarks. The first row shows the final data matrix *A* and the second row shows the final data matrix *B* and their respective decompositions. The first column is the data used as input into the JIVE algorithm. These final datasets were created by adding the second column representing the same joint structure shared between datasets, the third column representing the individual structure unique to each dataset, and the fourth column representing white noise.

We compared the runtimes of the original R.JIVE functions to the updated versions, which implement the changes in 2.1 to assess overall improvements. Two matrices *A* and *B* of size 200×1000 were generated from a common joint structure matrix, two unique individual structure matrices, and two residual error matrices generated from a standard normal distribution. A visualization of these datasets can be seen in Figure 1.

The first and second row contain the final matrix in the first column, the joint matrix in the second column, the individual matrix in the third column, and the error matrix in the fourth column for *A* and *B*, respectively. Matrix values range from between ∼–2 (represented by the color blue) and 2 (represented by the color red). A benchmark was performed in which both the old R.JIVE implementation and the updated version were repeated 20 times to record runtimes. We used given ranks of 1 for the joint structure and each individual structure.

All benchmarks were performed on a laptop with a 3.20 GHz AMD Ryzen 7 processor with 16.0 GB RAM using R version 4.2.2.

### 2.2 scRNA-seq datasets

One simulated dataset and two real scRNA-seq datasets were used to evaluate the performance of the batch correction methods. The two real datasets were acquired using the scRNAseq R package (Risso and Cole 2022). Principal variance component analysis (PVCA) (Li *et al*. 2009) was performed for each raw dataset to get an estimation of how much variability is attributed to batch effects, cell type effects, and random error. PVCA uses principal components from PCA and variance components analysis to fit a mixed linear model using the factors of interest as random effects to partition the total observed variability into variability due to batches, cell types, or residual error. This helps to quantify the impact of each source of variation on subsequent analyses.

#### 2.2.1 Simulated data

Simulated data were generated using the Splatter R package (Zappia *et al*. 2017), which allows the user to implement a Splat model. Under each of the simulation conditions, we simulated data for 5000 genes with two batches containing 1000 total cells. The simulation also consisted of three different cell types in similar proportions between batches. There were 100 unique simulation conditions, consisting of five levels of differential expression between cell types (location parameter of 0, 0.25, 0.5, 0.75, and 1), 5 levels of batch effects (location parameter of 0, 0.25, 0.5, 0.75, and 1), balanced and unbalanced cell type proportions, and balanced and unbalanced batch proportions.

For the JIVE method, we used a fixed set of ranks (joint rank of 10, individual ranks of 20) to reduce computation time. For Harmony, we adjusted some of the parameters (sigma = 2, lambda = 0.1) to improve performance in settings with large batch effects.

#### 2.2.2 Bacher T-cell data

The Bacher CD4+ T-cell RNA sequencing data (Bacher *et al*. 2020) were obtained from 6 unexposed and 14 COVID-19 patients. There are a total of 15 different batches and 6 different cell clusters provided. These data were chosen as there are not very large distinctions between batches and clusters, so we wished to see how the methods would perform in this type of scenario. The frequency of cells in each batch and cell group can be seen in Table 1.

**Table 1.** Batch and cell type frequency for Bacher T-cell data.

Batch Name	Central Memory	Cycling	Cytotoxic /Th1	Tfh-like
14	230	1	76	250
15	239	4	143	425

**Batch**	**Transitional**	**Type-1 IFN**	**Total**	

**Name**	**Memory**	**signature**		
14	190	7	754	
15	289	13	1113	

#### 2.2.3 Zilionis mouse lung data

The Zilionis mouse lung data (Zilionis *et al*. 2019) analyzed tumor-infiltrating myeloid cells in mouse lung cancers. There are a total of three different batches and seven different cell clusters provided. These data were chosen as there is some distinct separation due to a batch effect and the cell clusters are well separated. The frequency of cells in each batch and cell group can be seen in Table 2.

**Table 2.** Batch and cell type frequency for Zilionis mouse lung data.

Batch Name	B cells	T cells	Total
round1_20151128	641	579	1220
round2_20151217	879	434	1313

In this study, we evaluate the performance of the three batch-effect correction methods above across three scRNA-seq datasets with four different evaluation metrics. We also present multiple improvements to the JIVE computation algorithms, which help significantly decrease runtime compared to the current implementation in the R.JIVE R package.

### 2.3 Batch correction evaluation metrics

We employed four metrics to evaluate the performance of each of the batch correction methods: visual inspection of t-distributed stochastic neighbor embedding (t-SNE) (Van der Maaten and Hinton 2008), KNN batch effect tests (kBET) (Büttner *et al*. 2019), average silhouette width (ASW) (Rousseeuw 1987), and local inverse Simpson’s index (LISI) (Korsunsky *et al*. 2019). For the visual inspections, we expect to see cells from different batches overlapping each other in the plots with distinct cell type clusters. This is indicative of well-mixed (i.e. integrated) batches that preserve cell type heterogeneity.

While dimension reduction plots are a popular method for evaluating the performance of scRNA-seq batch-effect correction, any conclusions made from them are subjective. We include three numeric evaluation metrics to provide an objective sense of the performance for the three methods.

For each of the quantitative metrics, the inputs were based on dimension reduction of the batch-corrected data. For the raw data, Seurat v5, and Combat-seq, the inputs were the principal components of the batch-corrected data. For JIVE, Harmony, and Liger, the inputs were the implicit components from the batch correction in each method. Specifically for JIVE, we used the joint component scores for each cell.

### 2.4 t-Distributed stochastic neighbor embedding

t-SNE is a non-linear dimension reduction technique (Van der Maaten and Hinton, 2008) that aids in visualizing high-dimensional data by assigning each data point a location in a two or three-dimensional map. It aims to preserve as much of the local structure of the original data as possible while also revealing global structure such as clusters. High-dimensional Euclidean distances between points are used to create conditional probabilities of one point picking the other as its neighbor. A similar conditional probability is calculated for a low-dimensional representation of the data. The goal of t-SNE is to find a low dimensional (i.e. two or three dimensions) representation that matches the two probabilities as best as possible by minimizing a certain objective function. We performed t-SNE using the scater R package (McCarthy *et al*. 2017) version 1.26.1 and the Seurat R package (Stuart *et al*. 2019) version 4.3.0.

#### 2.4.1 KNN batch effect test

The kBET metric (Büttner *et al*. 2019) was constructed with the following premise in mind: a subset of a well-mixed dataset with batch-effects removed should have the same distribution of batch labels as the full dataset. A $\chi^{2}$-based test is performed for random subsets of a fixed size neighborhood, and results from each test (i.e. reject or fail to reject) is averaged over to provide an overall rejection rate. If the rejection rates are low, then we failed to reject most of the tests, and thus the distribution of batch labels in the small neighborhoods were not significantly different from the entire data’s distribution of batch labels. We performed kBET using the kBET R package (Büttner *et al*. 2017) version 0.99.6.

We calculate the rejection rates with neighborhood sizes equal to 5%, 10%, 15%, 20%, and 25% of the number of cells in each dataset. We then use the first 30 principal components from the batch-effect corrected datasets to perform the kBET at each neighborhood size. We then calculate the acceptance rate (1 – rejection rate) so that larger values are more desirable. The acceptance rates are then used for comparison across all methods.

#### 2.4.2 Average silhouette width

A silhouette is a measure of consistency within clusters of a given dataset (Rousseeuw 1987). For each data point in a given cluster, we calculate the mean distance between itself and all other points within the same cluster. We also calculate the smallest mean distance between itself and any other data point not in the same cluster. Then a silhouette is the difference of these two values scaled by the largest of the two. A silhouette takes on values between –1 and 1, with values close to 1 indicating that a particular point is appropriately clustered and values close to –1 indicating the opposite. The ASW is the average of all silhouette values, which gives a measure of how well-clustered the data are as a whole. We performed ASW calculations using the cluster R package (Maechler *et al*. 2022) version 2.1.4.

For our purposes, we use the Euclidean distance metric for all calculations. We then subsample our data down to 80% of the original and use the first 30 principal components from the subsampled batch-effect corrected datasets. We calculate two ASW metrics: ASW batch (the batch labels are the clusters) and ASW cell type (cell type labels are the clusters), and this process is repeated 20 times for each method. ASW batch and ASW cell type results from all methods are separately scaled to be between 0 and 1. We report 1 – ASW batch values so that large values are more desirable. The median values of each of these scores are then used for comparison across all methods.

#### 2.4.3 Local inverse Simpson’s index

The local inverse Simpon’s index (Korsunsky *et al*. 2019) first builds local Gaussian kernel-based distributions of neighborhoods around each cell. These neighborhoods are then used in conjunction with the inverse Simpson’s index to calculate a diversity score, which corresponds to the effective number of clusters in a particular cell’s neighborhood. We performed LISI calculations using the lisi R package (Korsunsky 2019) version 1.0.

We calculate two LISI metrics: LISI for batch label clusters (iLISI batch) and LISI for cell type clusters (cLISI cell type). Both LISI scores are calculated for each cell in the batch-effect corrected datasets for each method. iLISI and cLISI results from all methods are separately scaled to be between 0 and 1. We report 1 – cLISI cell type so that large values are more desirable. The median values of each of these scores are then used for comparison across all methods.

## 3. Results

### 3.1 JIVE runtime improvements

In base R, the SVD took ∼1.67 s on average. By comparison, the partial SVD functions only took 0.08, 0.12, and 0.15 s on average, respectively, for 1, 5, and 10 components. This is a 95.4% shorter runtime when estimating 1 component, a 93.1% shorter runtime when estimating 5 components, and a 91.0% shorter runtime when estimating 10 components. Using a partial SVD dramatically improves the runtime of the JIVE algorithm, since only *r* or $r_{i}$ components are needed in each SVD.

Performing matrix multiplication using precompiled C++ code also provided a sizable reduction in runtime. The original multiplication operator and the function using RcppArmadillo both averaged just under 0.3 s. The RcppEigen implementations utilizing 1, 2, 4, and 8 CPU cores had runtimes of 0.093, 0.047, 0.027, and 0.02 s on average, respectively. The difference between the %*% operator and the function implemented in RcppArmadillo were negligible. The function implemented in RcppEigen not only provided significant improvements over the base R operator, but it also allows for the user to specify the amount of CPU cores to utilize during runtime. We observed 67.5% shorter runtime when using the function with one core, a 83.2% shorter runtime when using two cores, a 90.6% shorter runtime when using four cores, and a 92.9% shorter runtime when using eight cores. Note that using a larger number of available CPU cores does not always provide an increase in speed. Computation on smaller matrices tend to be faster without using multiple cores, but computations on large matrices typically run faster on multiple cores.

With these two changes, the JIVE algorithm ran much faster. The original R.JIVE function performed the decomposition in ∼35.8 s on average, while the improved function completed it in ∼4.1 s on average. The two procedures produced close to identical results. Table 3 shows the proportion of variance attributable to joint structure, individual structure, and residual variance for the two methods.

**Table 3.** Proportion of variance attributed to JIVE decomposition.

Original	Data 1	Data 2	|	Updated	Data 1	Data 2
Joint	0.346	0.161	|	Joint	0.346	0.161
Individual	0.400	0.582	|	Individual	0.400	0.582
Residual	0.254	0.256	|	Residual	0.254	0.256

### 3.2 Simulated data

The only preprocessing step performed for the simulated data was log-normalization (McCarthy *et al*. 2017). Among the different simulation settings, the biggest difference in performance between the methods was in comparison of balanced versus unbalanced batches, so the following results have been split between the balanced and unbalanced batch settings.

The PVCA results for the simulation compared to the real datasets. are shown in Table 4. On average, the simulations have a similar breakdown of variability to the real datasets, but they have both stronger batch effects and weaker cell-type effects than the real datasets, making them even more challenging for batch correction. In the simulations, the batch effects contributed as much as 24.9% of the variability, which was under the following settings: cell-type location parameter of 0, batch location parameter of 1, balanced batch sizes, and balanced cell-type sizes. The cell effects contributed as much as 7.7% of the variability, which was under these settings: cell-type location parameter of 1, batch location parameter of 0.25, unbalanced batch sizes, and unbalanced cell-type sizes.

**Table 4.** Comparison of the variability due to batch effects, cell-type effects, and residuals among the three data sources used in this study, based on PVCA.^a^

Data Source	Batch	Cell	Residual
Simulation (Average)	0.078	0.027	0.895
Simulation 21	0.249	0.002	0.750
Simulation 85	0.016	0.077	0.907
Bacher T-cell	0.049	0.093	0.858
Zilionis Mouse Lung	0.023	0.113	0.863

a For the simulated data, values are averaged over the 100 iterations. The simulation settings with the highest proportion of batch effects (21) and cell effects (85), respectively, are also shown to illustrate the range of the simulation settings. The values shown are proportions; rows that do not add up to 1 are due to rounding.

Table 5 shows the results of the batch correction methods for the simulated data. Among the five methods, JIVE was slightly worse than most of the methods for removing batch effects under simulation conditions with balanced batches. Harmony performed much better than other methods in terms of kBET acceptance rate and LISI index, but most of the methods were relatively close in terms of ASW. But in terms of preserving cell-type effects, JIVE performed consistently better than the other four methods, in terms of both ASW and LISI. In terms of the LISI index, the performance of Liger was close to JIVE. But in terms of ASW, JIVE was consistently much better than the other methods. Overall, JIVE was competitive with other methods for batch correction, but preserved cell-type effects better than the other methods.

**Table 5.** Batch effect correction comparison averaged over 32 simulation settings with balanced batches (500 cells in each batch), excluding settings where batch effect or cell type effects are zero.^a^

Method	kBET Acceptance	1 - ASW Batch	ASW Cell Type	LISI Batch	1 - LISI Cell Type
Raw Data	0.000	0.634	0.104	0.006	0.866
JIVE	0.250	0.881	**0.290**	0.323	**0.935**
Seurat	0.317	0.976	0.158	0.462	0.878
Harmony	**0.708**	0.999	0.142	**0.790**	0.880
Liger	0.428	0.922	0.158	0.449	0.925
Combat-seq	0.104	**1.000**	–0.018	0.400	0.682

a Higher values indicate better mixing of different batches or better retention of cell type effects. The highest average value for each metric is in bold. kBET results are based on a 5% sample size; results were similar at larger sample sizes.

Table 6 shows the batch correction results with unbalanced batches. This is an area where the JIVE method did not perform well. With unbalanced batches, the batch effects of the larger batch are probably a larger proportion of the overall variability, resulting in these effects being classified as joint variability, even though they only explain variability in one batch. Fortunately, all of these methods are typically used with known batches, so it should generally be known whether the batches are balanced or unbalanced *a priori*. Harmony, the other PCA-based method, also performed worse with unbalanced batches, although the difference in performance was not as dramatic. The other methods were not as heavily impacted by unbalanced batches. If preserving cell types is important, than Liger is probably the best choice when the batches are unbalanced.

**Table 6.** Batch effect correction comparison averaged over 32 simulation settings with unbalanced batches (800 cells in Batch 1 and 200 cells in Batch 2), excluding settings where batch effect or cell type effects are zero.^a^

Method	kBET Acceptance	1 - ASW Batch	ASW Cell Type	LISI Batch	1 - LISI Cell Type
**Raw Data**	0.000	0.634	0.123	0.000	0.874
**JIVE**	0.000	0.221	0.131	0.000	0.883
**Seurat**	0.304	0.936	0.151	**0.290**	0.888
**Harmony**	**0.485**	0.887	0.149	0.119	0.888
**Liger**	0.385	0.916	**0.159**	0.260	**0.928**
**Combat-seq**	0.192	**1.000**	−0.021	0.119	0.696

a Higher values indicate better mixing of different batches or better retention of cell type effects. The highest average value for each metric is in bold. kBET results are based on a 5% sample size; results were similar at larger sample sizes.

In the remaining simulations, either the batch effect or the cell-type effect was set to zero. In the simulations where the cell-type location parameter was 0, none of the methods were able to appropriately separate cell types, as expected. When the batch effect location parameter was 0, most of the methods mixed the batches well. However, JIVE erroneously separated batches in this case, likely because the fixed ranks were overspecifying batch effects in this context.

### 3.3 Assessments using real data

#### 3.3.1 Bacher T-Cell data

Data preprocessing for the Bacher T-cell data consisted of log-normalization (as described for the simulated data), and the top 2000 genes with the highest variability across all batches were selected for analysis. This was performed using a standard workflow suggested in the Seurat R package. We also chose to select only two of the fifteen total batches. This reduced our total dataset size from 33538×104417 to 2000 genes by 1867 cells. The PVCA results for the Bacher T-cell dataset can be seen in Table 4.

**Figure 2.**
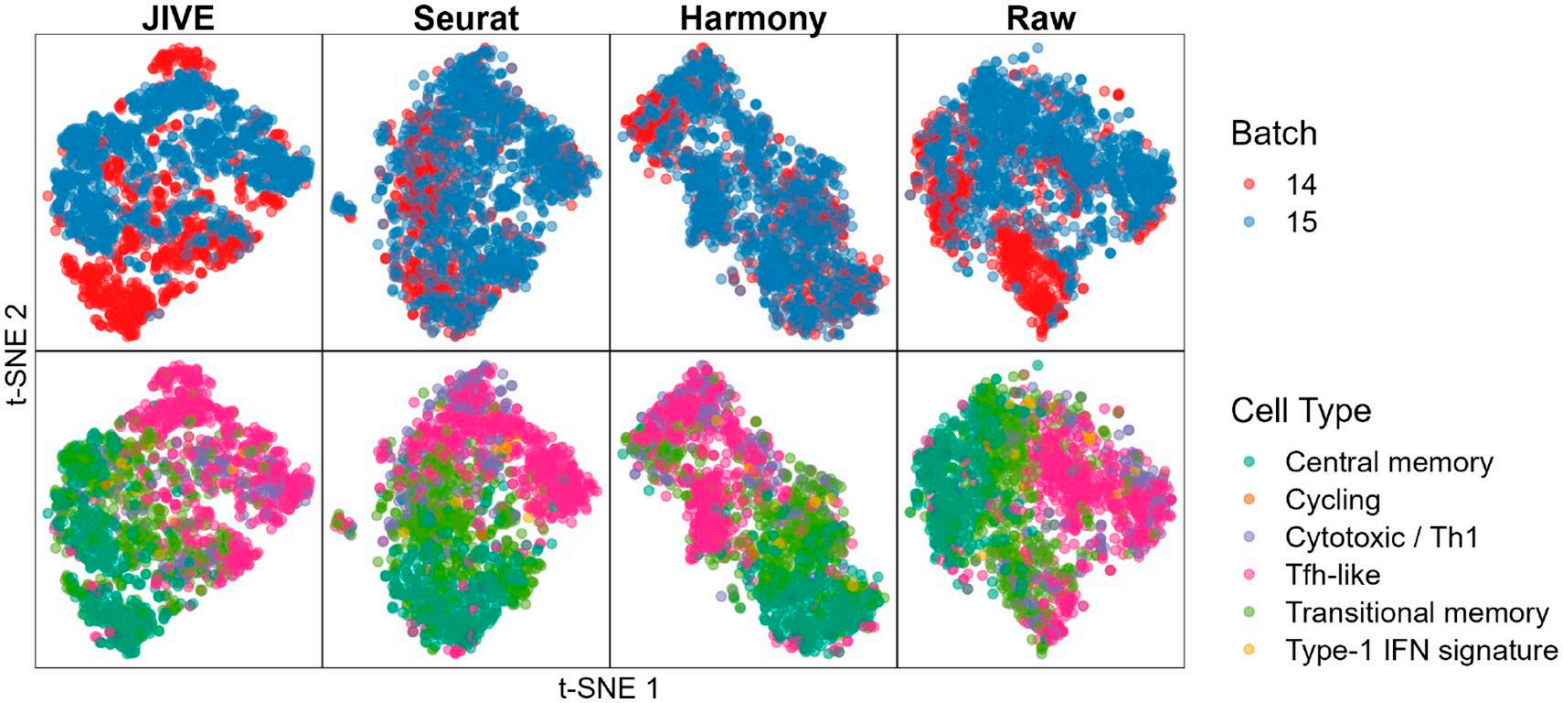
Qualitative evaluation of JIVE, Seurat, and Harmony batch-effect correction methods using t-SNE plots for the Bacher T-cell data. Each column represents a different method, with the fourth having no batch-effect correction applied. The first row has cells colored by batch and the second row has cells colored by cell type.

The variability attributed to the batch and cell clusters is ∼4.9% and 9.3%, respectively. The t-SNE plots can be seen in Figure 2.

**Figure 3.**
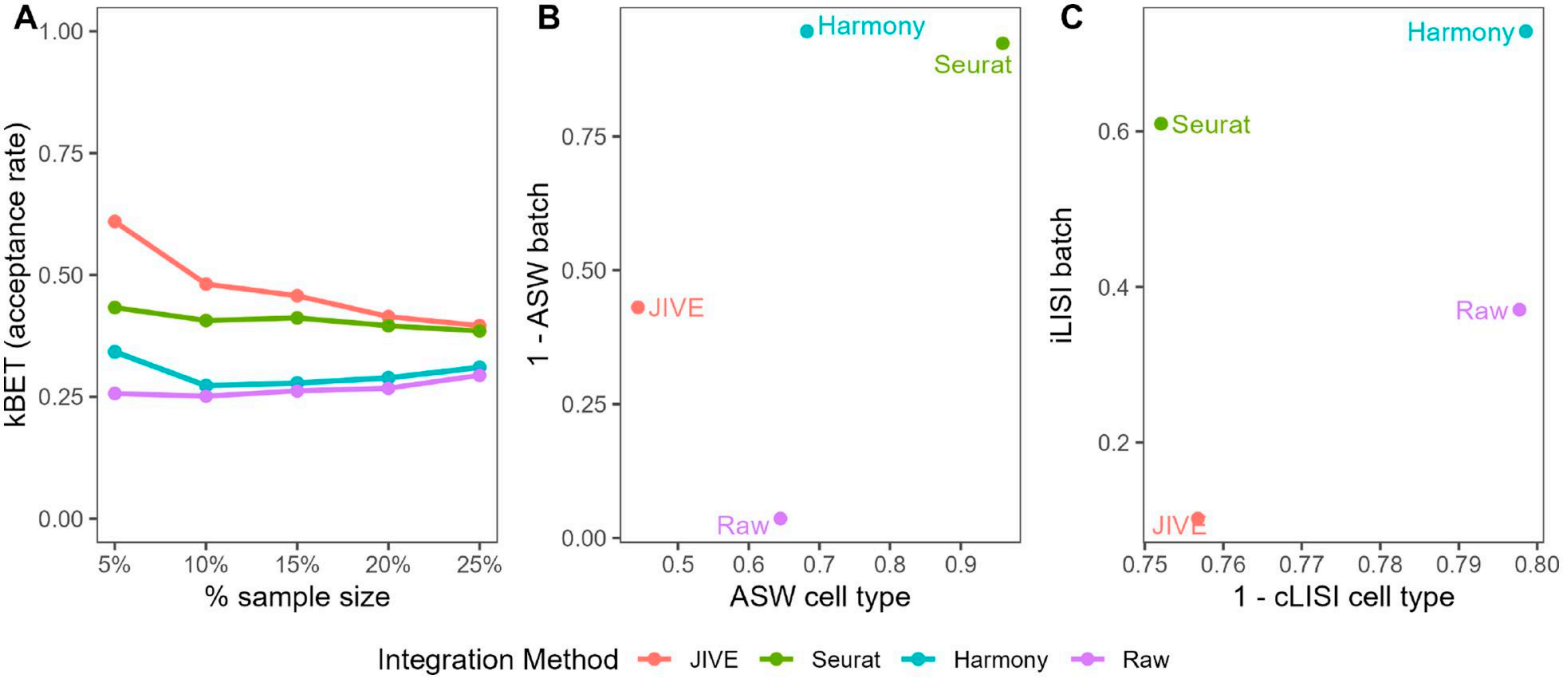
Quantitative evaluation of JIVE, Seurat v5, and Harmony batch-effect correction methods using (A) kBET, (B) ASW, and (C) LISI for the Bacher T-cell data. Methods with higher kBET acceptance rates performed best. Methods in the top right of the ASW and LISI plots performed best.

We see that Seurat and Harmony both have well-mixed batches in the dimensionality reduction plots, while JIVE does not. The cell clusters look to be well preserved in all three methods. The numeric evaluation metrics can be seen in Figure 3.

JIVE performs the best with regards to kBET, with Seurat v5 close behind. The acceptance rates for Harmony are just slightly higher than the raw data. Harmony and Seurat both perform best in the ASW metrics, while JIVE only outperforms the raw data with regards to ASW batch and actually performs worse in ASW cell type. Harmony is the clear winner in the LISI metrics followed closely by Seurat. Notably, JIVE performs worse than the raw data in both metrics. It is worth noting that the cLISI cell type is on a much tighter scale than the iLISI batch metric in the plot, so the perceived differences are not as large as they appear.

#### 3.3.2 Zilionis mouse lung data

We perform the same preprocessing as described for the Bacher T-cell data, select only two batches, and this time selecting only two cell types/clusters (B-cells and T-cells). This helps to reduce the data dimensions from 28205×17549 to 2000 genes by 2533 cells. The PVCA results for the Zilionis Mouse Lung dataset can be seen in Table 4.

**Figure 4.**
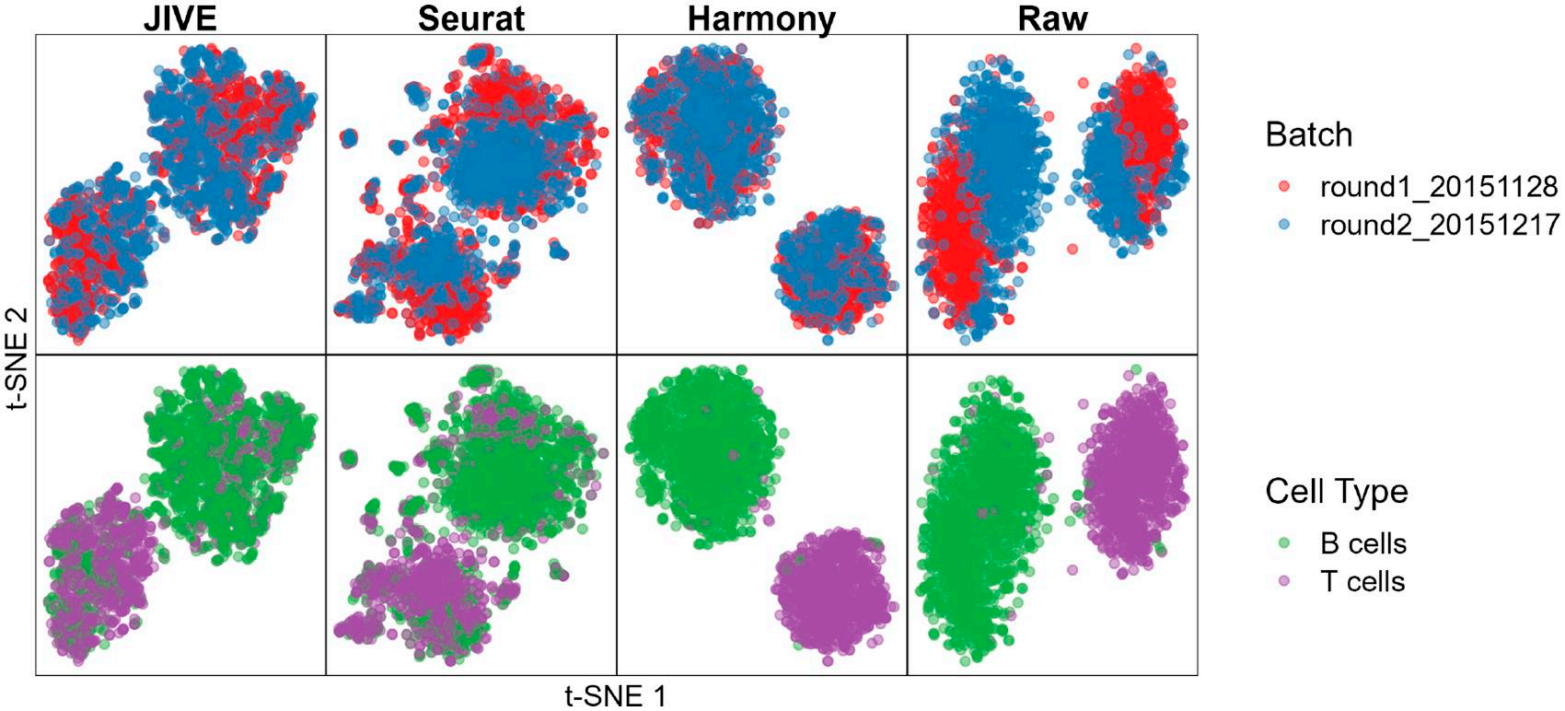
Qualitative evaluation of JIVE, Seurat, and Harmony batch-effect correction methods using t-SNE plots for the Zilionis mouse lung data. Each column represents a different method, with the fourth having no batch-effect correction applied. The first row has cells colored by batch and the second row has cells colored by cell type.

We see a similar breakdown of total variability as the Bacher T-cell data except a bit more is attributed to the cell clusters. The t-SNE plots can be seen in Figure 4.

**Figure 5.**
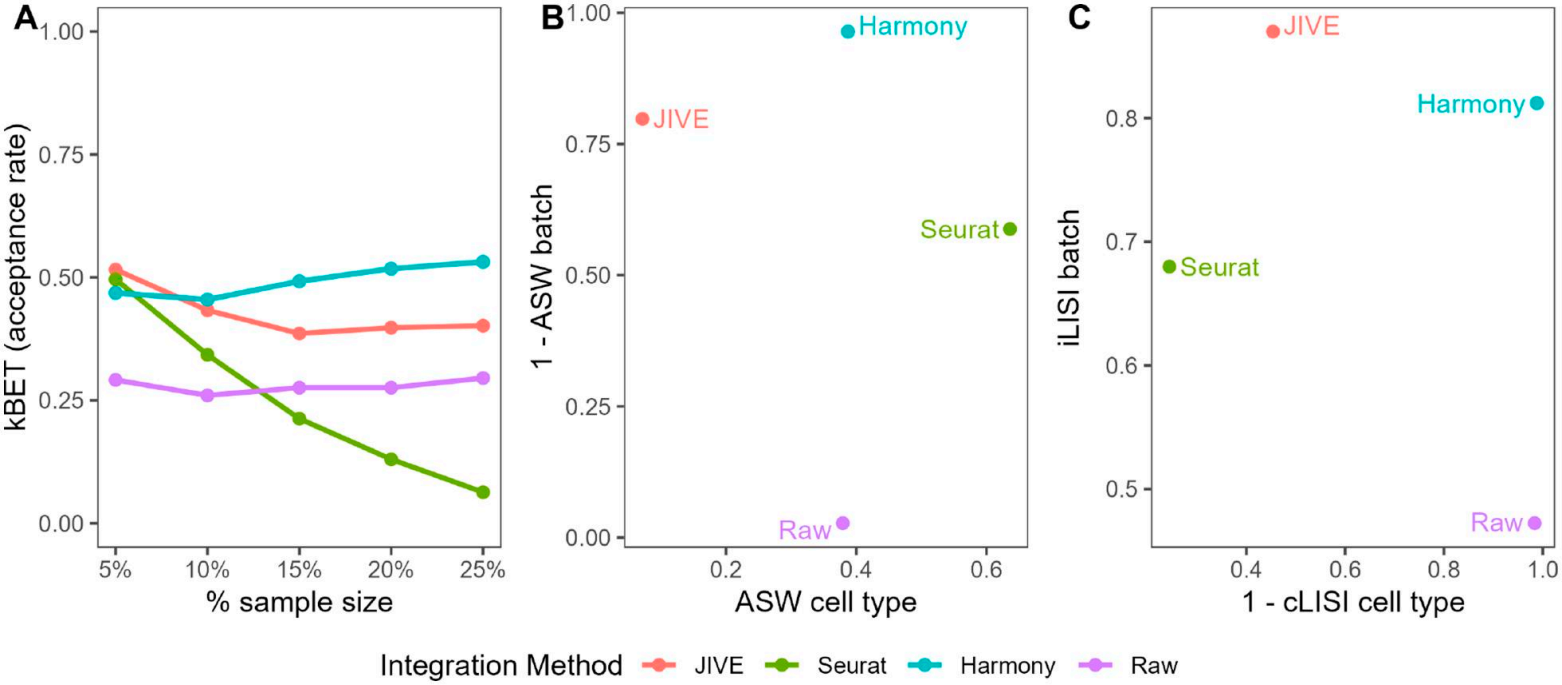
Quantitative evaluation of JIVE, Seurat, and Harmony batch-effect correction methods using (A) kBET, (B) ASW, and (C) LISI for the Zilionis mouse lung data. Methods with higher kBET acceptance rates performed best. Methods in the top right of the ASW and LISI plots performed best.

We see that each method has well-mixed batches and cell clusters are preserved. It is interesting to note that both JIVE and Seurat produced two distinct clusters that consist of a mix of both cell clusters, while Harmony was able to keep the cell clusters away from each other. The numeric evaluation metrics can be seen in Figure 5.

We see that all three methods perform similarly at low neighborhood levels. However, as the size increases, Seurat drops off dramatically. Harmony performs best at kBET with JIVE close behind. The ASW metric performances are mixed: each method is better than another at either ASW batch or ASW cell type. Harmony performs best at ASW batch, followed by JIVE and then Seurat. Seurat performs best at ASW cell type, followed by Harmony and then JIVE. In terms of batch correction, JIVE and Harmony are the best performers in the LISI metrics, with JIVE being the best with regards to LISI batch and Harmony winning out in LISI cell type. Seurat performs worse than both JIVE and Harmony, and only beats the raw data with regards to LISI batch. However, JIVE and Seurat v5 failed to preserve the cell type distinctions, with both methods having considerably worse LISI cell type values than the raw data.

## 4 Discussion

In this paper, we explored if the JIVE method was effective at performing batch-effect correction in scRNA-seq data. JIVE performed comparably or even better to existing methods in terms of batch correction metrics under many of the simulation conditions, especially with respect to preserving cell-type effects. However, the most important advantages that JIVE has are with interpretation. Many other batch correction methods, including Seurat and Harmony, involve some clustering to group the cell types better. This has advantages for identifying cell types, but it can also distort the original data if clustering cell types is not the end goal. In contrast, as a dimension reduction method, JIVE provides a direct mapping between the original data and the batch-corrected data. This makes it easier to interpret the JIVE results and use the batch-corrected data for further analysis compared to other methods.

Initially, the use of JIVE for this purpose was impractical due to its prohibitively long runtimes with data on the scale commonly seen in scRNA-seq datasets. The significant increase in speed due to the improvements we made to the algorithm makes it a much more realistic tool to use for batch-effect correction.

Even with these computational improvements, it is important to note a couple of limitations. First, because the JIVE improvements are reliant on using parallel processing, the computation time for the improved algorithm is heavily dependent on having access to multiple cores. In the simulation study, since the simulations were run in parallel, each iteration of JIVE was run on only a single core, and the computation time was about 6 h per iteration. Second, the rank-selection process can be very slow, since it requires running the model for each permutation of the data. Although we did not conduct benchmarks at very large sample sizes, these computational challenges are likely to limit the scalability of JIVE to large samples. In that case, it is highly advisable to choose ranks ahead of time. In the simulations, we used a fixed set of ranks that was likely underspecified in most of the conditions, and JIVE was still competitive. With enough cores, it might be feasible to scale to hundreds of thousands of cells with given ranks.

An alternative computational option might be to use AJIVE (Feng *et al*. 2018). AJIVE is based on the JIVE model but does not use an iterative approach for the model fit. With similar computational optimizations, an implementation of AJIVE would be more scalable to high samples sizes than JIVE. Using one of the scenarios from our simulation study, we tested the computational performance of JIVE compared to an existing Python implementation of AJIVE from the mvdr library (Carmichael 2020). The AJIVE algorithm was considerably faster, taking only 18 s to fit the model, compared to JIVE, which took slightly over 2 h. AJIVE was also more memory efficient, using 601 MB of memory compared to the 1180 MB used by JIVE. However, the rank-selection method from the AJIVE package consistently selected a joint rank of 0. This is a potential area for future work.

We expected JIVE to provide a more flexible alternative to other commonly used methods because not only does it allow the user to specify the ranks chosen for the low-rank approximations, but also primarily because it estimates joint structure and individual structure simultaneously. This simultaneous estimation procedure means that JIVE performs both batch correction and dimension reduction at the same time. This is preferred compared to other batch-effect correction methods because less information is lost: any effects not captured in the joint structure will be present in the individual structure, and vice versa. This implies that it is possible to reconstruct the original data using these two estimated structures. This approach differs from Seurat and Harmony where batch correction is performed first and then dimension reduction second. Some information is lost during the batch correction step because only the corrected datasets are estimated and everything else is discarded. One other potential advantage of the simultaneous estimation in JIVE is that one could theoretically use the individual structures as the basis for QC measures to evaluate whether technical effects were truly removed from the joint structure.

There are many other data integration methods, which we did not consider in this study. One network-based approach is Similarity Network Fusion (Wang *et al* 2014) where multiple sources of data are combined into a single network. It starts by constructing similarity networks for each data source independently and combines them by using a weighted averaging scheme to emphasize that edges are preserved across all networks. It then applies a clustering algorithm to identify groups of nodes that are highly interconnected and likely to be related. A Bayesian-based approach is Multi-Omics Factor Analysis (Argelaguet *et al*. 2018), which uses a probabilistic Bayesian and factor anaysis framework to decompose multi-omics data into shared and dataset-specific components. The shared factors are then interpreted in terms of biological processes and their relevance is evaluated.

### 4.1 JIVE batch-effect correction performance

In each scRNA-seq dataset, we tested the performance of each batch correction method on their ability to mix batches while still preserving the purity of the cell types/clusters. A commonly used method for evaluating batch integration is by visual inspection of dimension reduction plots, with the most common being PCA, t-SNE, or UMAP plots (Van der Maaten and Hinton 2008, McInnes *et al*. 2018, Tran *et al*. 2020). This method works well for simple cases like in the simulated data where the batch effects and cell clusters are clearly defined. However, this subjective method tends to become more difficult if there is not clear separation between batches or when cell types are very similar, as is the case in the Bacher T-cell data. This ambiguity that stems from visual inspection is the reason we employed the use of three numeric evaluation metrics to objectively assess the performance of each method. Note that while objective measurements are useful, we still believe that visual inspection can still provide useful insight during exploratory analysis. Note that none of the metrics simultaneously test both the quality of batch mixing and preservation of cell types, and the development of such a metric would be of great interest.

Overall, Harmony performed the best in the simulated data and Zilionis mouse lung data, where batch effects were distinct and cell type effects were large. It consistently performed well on kBET and LISI metrics that take into account the structure of each cell’s local neighborhood. The t-SNE plots were also consistent with its performance in the numeric metrics. JIVE performed second best with regards to metrics concerning batch mixing, but it struggled with cell type purity metrics in this dataset. It is not surprising that JIVE performed reasonably well on this dataset, since the batches were relatively balanced (1220 and 1313 cells, respectively). This is consistent with the simulations, where JIVE performed better under balanced batches. However, one major difference from the simulated data is that JIVE was better at preserving cell-type effects in the simulated data but not as effective at eliminating batch effects. But in the Zilionis mouse data, JIVE was very effective at removing batch effects but was not effective at retaining cell-type effects. This difference may be due to the correlation between the batch effects and cell-type effects. One limitation of JIVE is that there is a potential risk of losing biological signals when biological factors and batch factors are confounded. This is especially true if the joint rank is under-specified in the JIVE decomposition, as any biological effects that are not captured by the joint structure can then be captured in the individual structure, erroneously treating it as batch effects.

JIVE did not perform well in the Bacher T-cell data. The most likely issue for JIVE in these data was the unbalanced batches, which were also a problem in the simulation study. This dataset also contained multiple cell types with heavy overlap, making it a challenging dataset overall. Based on ASW, Seurat v5 was the best method for this dataset, but Harmony was best in terms of LISI. Interestingly, despite poor performance in the other metrics, JIVE did have the highest kBET acceptance rate for this dataset.

Based on our results, we recommend using JIVE only in scenarios where the batch and cell effects are not highly correlated, and the batches are balanced.

### 4.2 Limitations and future work

Our results showed that there is potential for multi-source dimension reduction techniques to be effective at correcting for batch effects. The major advantage of JIVE over existing methods is that it has a very simple interpretation. However, a major limitation of the JIVE decomposition is that it is best suited to data that are normally distributed. In contrast, scRNA-seq data tends to be zero-inflated and skewed. Therefore, an alternative method that can account for different distributions of data or zero-inflation may be more appropriate. There are some recent methods that can incorporate different distributions. A generalized association study (GAS) is an extension of JIVE that allows for exponential family distributions (Li and Gaynanova 2018). A GAS model with a negative binomial link might be a better fit to the scRNA-seq data. Another option would be simultaneous non-Gaussian component analysis, which divides the resulting components into Gaussian and non-Gaussian components (Risk and Gaynanova 2021). However, more research is needed to develop multi-source dimension reduction methods that can effectively account for zero-inflation.

Another limitation is the narrow scope of datasets used in this study. We only compared three datasets and considered a maximum of two batches at a time with at most six cell types. Subsets of batches in the real datasets were chosen for the sake of simplicity; however, in practice, it may be of interest to integrate datasets from much more than two experiments or batches. A more comprehensive set of 10 different datasets was analyzed in Tran *et al*. (2020), including 5 distinct scenarios: identical cell types sequenced by different technologies, non-identical cell types across batches, data from more than two batches, big datasets (>100,000 cells each), and multiple simulated datasets. Assessing the performance of JIVE in a wider range of data scenarios would help give a better sense of its ability to perform batch-effect correction.

One condition where JIVE did not perform well was with unbalanced batches. In this case, a lot of the batch effects are likely being absorbed into the joint components. There are multiple possible adjustments that might alleviate this issue. One solution would be subsampling from the larger batches to get equal sample sizes in each batch. Another option might be to scale the datasets relative to their size, so that larger batches do not contribute a disproportionate amount of the total variability.

Another limitation is that JIVE generally requires complete data. However, it is possible that gene sets will not match between batches in real datasets. Although the R implementation includes a preprocessing step to impute missing data, this is not likely to work well if genes are entirely missing from a batch. If gene sets differ, we recommend using the intersection of the gene sets.

Missing data are an important area for future work. It would be ideal to have a better option than taking the intersection of gene sets when the gene sets do not match between batches. Additionally, it is common to have a high proportion of zeros in single-cell sequencing, which is not something that JIVE is well equipped to handle. These zeroes may be due to biological sources (genes not expressed in a cell) or technical sources (genes not expressed at a high enough level to be detected). It may be possible to modify the JIVE method to use a zero-inflated model instead of the standard PCA-based model to better accommodate this feature of the data.

Key Points

- JIVE was originally developed for bulk sequencing data, but this R implementation is much more computationally efficient than the previous one to accommodate the higher computational needs of single-cell RNA-sequencing data.
- JIVE offers a more intuitive alternative to existing batch correction methods, since it decomposes the variability into variability within batches and variability shared across batches.
- JIVE is competitive with existing batch-correction methods when batch sizes are balanced and the correlation between batch effects and cell-type effects is small.
- There is promise for multi-source dimension reduction methods in batch-effect correction, but future methods that incorporate skewed distributions or zero-inflation may improve the performance considerably.

## Data Availability

The JIVE implementation used for this analysis can be found at https://github.com/oconnell-statistics-lab/scJIVE. The full code used to perform this analysis can be found at https://github.com/jwhastings/scProject/tree/main. This includes code to replicate the simulated data and to read and process the Bacher T-cell data and the Zilionis mouse lung data.
